# Transcriptional Analysis of *Lactobacillus brevis* to N-Butanol and Ferulic Acid Stress Responses

**DOI:** 10.1371/journal.pone.0021438

**Published:** 2011-08-02

**Authors:** James Winkler, Katy C. Kao

**Affiliations:** Department of Chemical Engineering, Texas A&M University, College Station, Texas, United States of America; Naval Research Laboratory, United States of America

## Abstract

**Background:**

The presence of anti-microbial phenolic compounds, such as the model compound ferulic acid, in biomass hydrolysates pose significant challenges to the widespread use of biomass in conjunction with whole cell biocatalysis or fermentation. Currently, these inhibitory compounds must be removed through additional downstream processing or sufficiently diluted to create environments suitable for most industrially important microbial strains. Simultaneously, product toxicity must also be overcome to allow for efficient production of next generation biofuels such as n-butanol, isopropanol, and others from these low cost feedstocks.

**Methodology and Principal Findings:**

This study explores the high ferulic acid and n-butanol tolerance in *Lactobacillus brevis*, a lactic acid bacterium often found in fermentation processes, by global transcriptional response analysis. The transcriptional profile of *L. brevis* reveals that the presence of ferulic acid triggers the expression of currently uncharacterized membrane proteins, possibly in an effort to counteract ferulic acid induced changes in membrane fluidity and ion leakage. In contrast to the ferulic acid stress response, n-butanol challenges to growing cultures primarily induce genes within the fatty acid synthesis pathway and reduced the proportion of 19∶1 cyclopropane fatty acid within the *L. brevis* membrane. Both inhibitors also triggered generalized stress responses. Separate attempts to alter flux through the *Escherichia coli* fatty acid synthesis by overexpressing acetyl-CoA carboxylase subunits and deleting cyclopropane fatty acid synthase (*cfa*) both failed to improve n-butanol tolerance in *E. coli*, indicating that additional components of the stress response are required to confer n-butanol resistance.

**Conclusions:**

Several promising routes for understanding both ferulic acid and n-butanol tolerance have been identified from *L. brevis* gene expression data. These insights may be used to guide further engineering of model industrial organisms to better tolerate both classes of inhibitors to enable facile production of biofuels from lignocellulosic biomass.

## Introduction

*Lactobacillus brevis*, a fastidious heterofermentative lactic acid bacteria often found in fermentation processes [1], [2], possesses a multitude of industrially advantageous complex phenotypes, including tolerance of short-chain alcohols such as ethanol and n-butanol [3]–[5], aromatic organic compounds [6], and hops [7]. However, given that *L. brevis* has only recently begun to be extensively characterized [8]–[13], little is known about the molecular mechanisms responsible for the array of environmental tolerances displayed by *L. brevis*. Of particular interest is the tolerance of *L. brevis* to phenolic compounds that are generated during the preparation of lignocellulosic biomass for downstream use [14]–[18]. Several studies have examined the high phenolic acid tolerance of lactic acid bacteria involved in wine making or other fermentation processes [19]–[23] and have consistently identified these compounds as inhibitory at high concentrations. *L. brevis* in particular has been shown to possess superior tolerance of phenolic compounds compared to other lactic acid bacteria [6], [21]. An improved understanding of the mechanism of phenolic compound tolerance in *L. brevis* would therefore be an important step forward in the industrial use of cellulosic biomass.

One of the most common model compounds used to screen for phenolic compound tolerance is ferulic acid [6], [24], [25], due to its toxicity and abundance in biomass. This compound is ubiquitous in plant cell walls, providing both mechanical strength and rigidity [26], [27]. Chemical or enzymatic treatments of lignocellulosic biomass therefore release ferulic acid in solution as a byproduct of processing [28], [29]. Ferulic acid is one of the most toxic hydroxycinnamic acids [22], [23], causing complete inhibition of *C.beijerinckii* growth at 2 g/L [30]. Phenolic acids may also damage intracellular hydrophobic sites and cause ion leakage by altering membrane permeability [25], [31], [32] leading to cell death at high concentrations. The use of cellulosic biomass consequently requires microbes with intrinsically higher tolerance of phenolic compounds or extensive downstream processing [33]–[35]. Given the chemical similarity of ferulic acid to most phenolic acids present in cellulosic biomass derivatives and its abundance in biomass, tolerance of ferulic acid can potentially be an useful indicator of how a given organism will tolerate the other phenolic compounds generated during biomass processing.

Though biomass hydrolysates can be used as the feedstock for many biotechnological processes, their use in the production of chemicals such as ethanol, n-butanol, and others is attracting much attention as a result of their low cost and independence from food crops [36]–[38]. The toxicity of the hydrolysates and the various fuel alcohols themselves renders efficient production of these compounds from unprocessed biomass challenging [39]. Production of n-butanol is particularly desirable due to its chemical similarity to existing petrochemical fuels and a superior energy content compared to ethanol [40], [41]. However, n-butanol tends to partition into lipid membranes due to its low partition coefficient 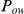 [42], [43], triggering changes in membrane fatty acid composition [44], [45] and deleterious effects on cell metabolism due to the chaotropic properties of this alcohol [42]. Previous studies have shown that the 3% (v/v) n-butanol tolerance of *L. brevis* exceeds that of most other strains [3]; understanding the mechanisms that confer n-butanol tolerance in *L. brevis* may simplify efforts to engineer this phenotype into industrial strains suitable for large-scale biological n-butanol production. A strain tolerating both biomass inhibitors and n-butanol simultaneously would be advantageous for the economical production of n-butanol. Numerous studies have attempted to address these roadblocks to n-butanol production by characterizing n-butanol tolerance limits and mechanisms in *Escherichia coli* [46], *Clostridium acetobutylicum* [47]–[51], and other organisms [52], [53] to enable the creation of strains suitable for the economical production of n-butanol on a large scale. Given its multiplicity of nutrient auxotrophies and slow growth rate, *L. brevis* itself is unlikely to serve as a cost-effective host for most bioprocesses despite having been successfully engineered for n-butanol production [54]. However, an understanding of the basis for the *L. brevis* phenolic and n-butanol tolerance phenotypes could provide new insight on how to engineer these desirable characteristics in organisms more amenable to genetic manipulation and industrial usage.

To that end, this work presents the first transcriptional analysis of *L. brevis* in response to phenolic acid and n-butanol stresses. Given the undesirability of using *L. brevis* as a host for bioprocesses for the reasons previously outlined, the primary goal of this study is to identify possible mechanisms for n-butanol and phenolic acid tolerance for subsequent study in other organisms such as *E. coli* that are more amenable to engineering efforts. In this manner the need to develop techniques necessary for facile engineering of *L. brevis* will be avoided. The transcriptional profiles under each inhibitor are shown to share elements of a generalized stress response, including the production of protein chaperones, increased transcription of genes involved in energy metabolism, and a general repression of growth related functions. However, ferulic acid challenge triggered transcription of a host of uncharacterized proteins containing transmembrane domains, while n-butanol challenge leads to increased expression of the fatty acid synthesis pathway over time. Several possible mechanisms explaining the transcriptional responses are proposed and in the case of n-butanol tolerance, tested with two *E. coli* strains with modified fatty acid synthesis pathways.

## Results and Discussion

The principal goal of this study was to identify mechanisms that may contribute to ferulic acid and n-butanol tolerance in *L. brevis* through the application of novel gene expression microarrays. Cultures in mid-exponential growth were separately challenged with both inhibitors and the transcriptional response monitored to identify genes that respond immediately to each insult, along with any long term adaptation over the experimental time course. Contrasting the transcriptional responses allows for the identification of genes that are likely expressed in response to general stressors (e.g. protein chaperones) and those genes that are specific to each stress condition, providing additional insight into how *L. brevis* reshapes its transcriptional program in response to environmental challenges.

### Transcriptional Response to Ferulic Acid Stress

**Figure 1:**
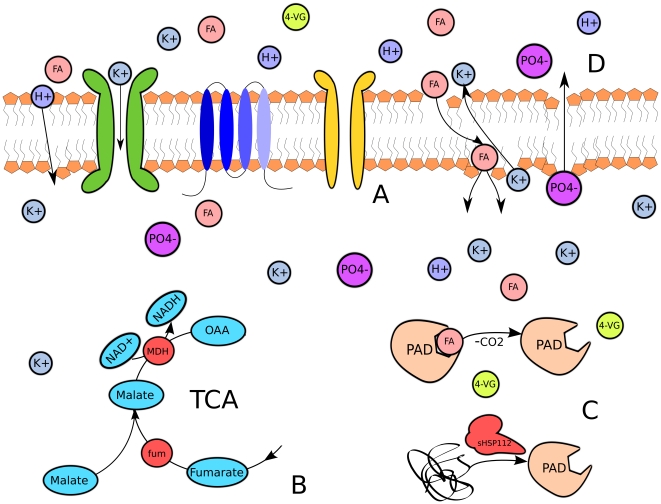
The essential *L. brevis* stress response to ferulic acid. **A.** Expression of many uncharacterized, multi-domained membrane proteins that may function as ion transporters, ferulic acid or 4-vinylguaicol exporters, among several possibilities. **B.** Upregulation of fumarase (fum), malate dehydrogenase (mdh), and malate permases involved in the citric acid cycle. NADH dehydrogenase and glutathione reducatase may also play a role in NAD+/NADH recycling and superoxide generation. **C.** Expression of small HSP to ensure proper folding of phenolic acid decarboxylase (PAD) which converts ferulic acid to 4-vinylguaicol. **D.** Representation of membrane damage and ion leakage triggered by ferulic acid induced lipid packing disruption.

Following the addition of 24 mM ferulic acid, the transcriptional data revealed an immediate stress response (summarized in Figure 1) in the *L. brevis* cultures. In all, the combined functional distribution of upregulated genes during both time points, in terms of their corresponding cluster of orthologous genes (COGs), demonstrated a marked departure from that of the reference samples. Those genes involved in carbohydrate transport and metabolism (COG G), transcriptional regulation (COG K), and amino acid transport and metabolism (COG E) appear to have undergone severe pertubations as *L. brevis* adjusts its transcriptional program to respond to the ferulic acid challenge. The functional distribution of differentially expressed genes is shown in Figure S1 and the expression levels of genes that appear most critical to the stress response are presented in Table 1; a complete list of significantly expressed genes is given in Table S1. Many up-regulated genes encode proteins involved in the citric acid cycle or sugar utilization, such as malate dehydrogenase, fumarase, catabolite control protein A (*ccpA*), or sugar transporters. The expression of a NADPH-quinone oxidoreductase may be an attempt to control excess 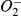 levels [55] generated within an aerobic environment. The up-regulated heat shock protein (LVIS-0112), conserved in many lactic acid bacteria species [56], may participate in folding the heat labile phenolic acid decarboxylase [57]–[60]; however, this chaperone is also expressed in response to n-butanol stress so it is likely a general stress response to protein mis-folding. The phenolic acid decarboxylase (LVIS-0213) of *L. brevis* is by far the most overexpressed gene, as expected given the role of this enzyme in degrading phenolic acids.

**Table 1:** Expression Levels of Select Significantly Upregulated Genes during Ferulic Acid Stress.

Locus	Gene Description	COG(s)	15 min Log 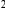	135 min Log 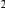
**COG C**				
LVIS-0076	NADPH-quinone oxidoreductase	C	0.12	**2.56**
LVIS-0714	Fumarate hydratase	C	0.95	**2.15**
LVIS-2203	Malate dehydrogenase	C	1.54	**2.24**
**COG G**				
LVIS-1730	Major facilitator superfamily permease	G	**2.88**	**3.41**
LVIS-1917	Major facilitator superfamily permease	G	**2.06**	**2.74**
LVIS-2254	ABC-type maltose transport system, permease	G	1.25	**2.96**
LVIS-2255	ABC-type sugar transport system, permease	G	**1.39**	**2.48**
LVIS-2256	ABC-type sugar transport system, periplasmic	G	**1.54**	**2.99**
**COG E**				
LVIS-0113	Amino acid transporter	E	**2.10**	**3.83**
LVIS-1879	Amino acid transporter	E	**1.51**	**2.78**
LVIS-1951	Glycine cleavage system H protein (lipoate-binding)	E	0.28	**2.60**
**COG IQ**				
LVIS-0213	Phenolic acid decarboxylase	Q	**5.75**	**4.19**
LVIS-0378	3-ketoacyl-(ACP) reductase (FabG)	IRQ	**1.52**	**2.48**
LVIS-0924	Biotin operon repressor	H	**1.77**	0.05
**COG KO**				
LVIS-0086	Peroxiredoxin	O	**2.51**	**2.22**
LVIS-0112	Molecular chaperone (sHSP)	O	**4.18**	**2.62**
LVIS-2147	Catabolite control protein A	K	**1.51**	1.27
**COG S**				
LVIS-0031	Hypothetical protein LVIS-0031	S	**1.34**	1.57
LVIS-0155	Hypothetical protein LVIS-0155	S	0.19	**2.13**
LVIS-0157	Hypothetical protein LVIS-0157	S	0.93	**2.61**
LVIS-0212	Hypothetical protein LVIS-0212	S	**4.26**	**3.93**
LVIS-0262	Hypothetical protein LVIS-0262	S	1.24	**2.36**
LVIS-0305	Hypothetical protein LVIS-0305	S	0.86	**2.81**
LVIS-0314	Hypothetical protein LVIS-0314	S	0.44	**2.58**
LVIS-0445	Hypothetical protein LVIS-0445	S	**1.90**	**2.23**
LVIS-0552	Hypothetical protein LVIS-0552	S	**1.32**	**2.54**
LVIS-0712	Hypothetical protein LVIS-0712	S	**1.87**	0.73
LVIS-1831	Hypothetical protein LVIS-1831	S	**1.48**	1.73
LVIS-1880	Hypothetical protein LVIS-1880	S	**2.84**	**2.91**
LVIS-1881	Hypothetical protein LVIS-1881	S	**2.64**	**2.12**
LVIS-2013	Hypothetical protein LVIS-2013	S	0.24	**2.22**
LVIS-2118	Hypothetical protein LVIS-2118	S	0.66	**2.17**
LVIS-2201	Hypothetical protein LVIS-2201	S	0.53	**2.99**
**COG P**				
LVIS-0472	ABC-type Mn2+/Zn2+ transport system, permease	P	0.56	**2.08**

COG definitions: C: Energy production and conversion, G-Carbohydrate transport and metabolism, I-Lipid transport and metabolism, K-Transcription, O-Posttranslational modification, protein turnover, chaperones, P-Inorganic ion transport and metabolism, Q-Secondary metabolites biosynthesis, transport and catabolism, and S-Function Unknown. COG S includes only those hypothetical proteins with one or more trans-membrane domains identified by the trans-membrane hidden Markov model tool TMHMM. Each log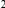 score is bolded if the gene is significantly overexpressed during that time point.

Many of the up-regulated genes detected encode hypothetical proteins with poor or no functional annotation, as is expected given the relative dearth of studies that have experimentally characterized *L. brevis* on a genetic level. Screening these *L. brevis* hypothetical ORFs using the trans-membrane hidden Markov model TMHMM [61] revealed that 28 of the 55 up-regulated hypothetical genes in either time point possess one or more transmembrane helices. These data suggest that the proteinaceous rather than lipid content of the membrane changes to counteract ferulic acid toxicity. These proteins may act as membrane stabilizers or ion transporters to counteract the membrane disruption triggered by ferulic acid; however, n-butanol exposure did not alter expression of these genes compared to the reference culture. These genes may therefore respond specifically to ferulic acid stress in particular or phenolic compound insults in general. Two uncharacterized major facilitator superfamily permeases (LVIS-1730 and LVIS-1917) with unknown substrate specificity were also overexpressed at different time points in response to ferulic acid stress; these exporters may be involved in the active export of ferulic acid or the decarboxylated product 4-vinylguaicol. These uncharacterized membrane proteins and transporters are promising targets for additional study to unravel the precise genetic basis of ferulic acid tolerance in *L. brevis*, especially given their unique occurrence in the ferulic acid stress profile.

In contrast to these shifts in energy metabolism, transcriptional regulation, and xenobiotic degradation, genes involved in membrane biogenesis (COG M) surprisingly comprise only 4% of the 195 genes, indicating that *L. brevis* may not appreciably alter the lipid composition of cytoplasmic membrane or the thickness of the outer peptidoglycan layer in response to ferulic acid. However, the consistent upregulation of 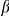-ketoacyl-(acyl-carrier-protein) reductase (*fabG*, LVIS-0378), a key enzyme in type II fatty acid synthesis [62], suggests that membrane lipid abundance may be altered as a possible defense mechanism against ferulic acid induced membrane fluidity. This hypothesis is supported by observed changes in the membrane of the closely related *Lactobacillus plantarum* when challenged with caffeic and ferulic acids [63]. Strangely, no other genes in the fatty acid synthesis pathway are upregulated so it is possible that the FA synthesis pathway in *Lactobacillus* is primarily regulated post-transcriptionally. Given the similarity between the organisms, membrane composition changes of *L. brevis* in response to ferulic acid are very likely to reflect those observed in the phenolic acid tolerant *L. plantarum*.

As expected, the highly reduced growth rate of *L. brevis* in the presence of ferulic acid is reflected in the functional roles of the down-regulated genes over the course of the challenge. An overview of the genes affected by ferulic acid stress is given in Table 2. Those genes encoding proteins responsible for amino acid and carbohydrate metabolism, transcription, and translation are strongly repressed, indicating that a metabolic shift from normal growth to a ferulic acid stress response has occured. Repression of several H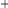 antiporters may be due to a disruption of the cell's normal proton gradient [64], [65]. A total of 35 genes encoding proteins without known functional roles were down-regulated, but only 8 of these proteins appear to contain trans-membrane segments. On the whole, genes repressed during the ferulic acid challenge are similar to those seen during the n-butanol challenges (see below), implying growth and certain types of nutrient assimilation are inhibited within stressful environments. This result is also consistent with the repression of two key proteins involved in cell division (LVIS-0848, LVIS-1402) and observed growth defects for both stress conditions.

**Table 2:** Expression Levels of Select Significantly Downregulated Genes during Ferulic Acid Stress.

Locus	Gene Description	COG(s)	15 min Log 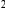	135 min Log 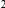
**COG C**				
LVIS-0199	Na 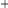 /H 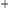 antiporter	C	**−2.19**	**−4.62**
LVIS-2211	Na 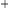 /H 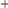 antiporter	C	−0.67	**−4.08**
LVIS-2141	NhaP-type Na 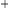 /H 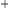 and K 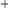 /H 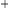 antiporter	P	**−2.01**	−0.51
**COG DS**				
LVIS-0848	Cell division initiation protein	D	**−1.76**	−1.32
LVIS-1455	Cell division protein MraZ	S	−0.39	**−2.97**
**COG E**				
LVIS-1587	Amino acid transporter	E	**−1.95**	**−3.15**
LVIS-2023	Carbamate kinase	E	**−1.84**	−1.09
LVIS-2024	Transaminase	E	**−1.94**	−0.85
LVIS-2025	Amino acid transporter	E	**−2.23**	−1.31
LVIS-2212	Amino acid transporter	E	−1.01	**−4.18**
LVIS-2213	Glutamate decarboxylase	E	−0.74	**−3.70**
**COG I**				
LVIS-0934	Acyl carrier protein	IQ	−0.24	**−2.54**
LVIS-0936	Transcriptional regulator	K	−0.58	**−2.66**
LVIS-0937	FabZ	I	−0.59	**−2.59**
LVIS-0954	Glycerol-3-phosphate acyltransferase PlsX	I	−0.65	**−2.24**
**COG S**				
LVIS-0064	Hypothetical protein LVIS-0064	S	**−1.97**	**−2.29**
LVIS-0422	Hypothetical protein LVIS-0422	S	**−2.56**	**−2.52**
LVIS-0899	Hypothetical protein LVIS-0899	S	**−1.74**	**−2.68**
LVIS-1369	Hypothetical protein LVIS-1369	S	**−1.68**	**−1.97**
LVIS-1834	Hypothetical protein LVIS-1834	S	**−1.77**	−0.83
LVIS-1895	Hypothetical protein LVIS-1895	S	−0.74	**−2.46**
LVIS-2216	Hypothetical protein LVIS-2216	S	**−2.25**	**−4.64**

COG definitions: C: Energy production and conversion, D-Cell cycle control, cell division, chromosome partitioning, P-Inorganic ion transport and metabolism, and S-Function Unknown. COG S includes only those hypothetical proteins with one or more trans-membrane domains identified by the trans-membrane hidden Markov model tool TMHMM. Each log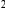 score is bolded if the gene is significantly overexpressed during that time point.

### Transcriptional Response to n-Butanol Stress

Given the likelihood that lignocellulosic biomass would be used for bio-butanol production, the transcriptional response of *L. brevis* to n-butanol insults was tracked over time in hopes of of revealing unique resistance mechanisms that could be combined with the insights gained from the ferulic acid challenges to produce a superior industrial strain. Unlike the gene expression pattern observed in the case of ferulic acid challenges, the addition of 1–2% n-butanol induced profound perturbations in lipid biosynthesis, protection against excessive oxidative stress via several means, and synthesis of protein chaperones. Given the similarity between the transcriptional profiles in response to 1% and 2% n-butanol, only the latter will be discussed here. A survey of the *L. brevis* n-butanol transcriptional response is provided in Tables 3 and 4, and the complete lists of up and down regulated genes are given in Tables S2 and S3. The functional distribution (in terms of COGs) over time is shown in Figure S2. Putative chaperones or chaperone components such as *dnaJ* (LVIS-1328), chaperone LVIS-0112, and ATP-binding subunits (LVIS-762, LVIS-1554, LVIS-1700) are significantly upregulated for the entire experimental time course. This pattern of chaperone was also similar to that observed during ferulic acid exposure, suggesting that these proteins are typically expressed as a result of environmental stress in general instead of being tied to a specific chemical stressor. There are several signs of oxidative stress as well; the continually increasing upregulation of two peptide methionine sulfoxide reductases (LVIS-0809 and LVIS-0810) which repair oxidative damage to methionine residues in conjunction with thioredoxin, NADPH-quinone oxidoreductase (LVIS-0076), and one thioredoxin (LVIS-1216) following n-butanol exposure. This response comports well with previous reports of n-butanol-induced oxidative stress in *E. coli* [46]. Another component of the *L. brevis* oxidative stress response is the accumulation of manganese due to its lack of traditional superoxide dismutases (as determined by BLAST) [66], [67]. The high expression of the Mn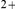/Zn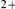 ion transport system (LVIS-0451 and LVIS-0452) over the time series therefore provides additional evidence that n-butanol triggers an oxidative stress response.

**Table 3:** Expression Levels of Select Significantly Upregulated Genes during 2% n-butanol Stress.

Locus	Gene Description	COG(s)	15 min Log 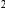	75 min Log 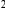	135 min Log 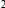
**COG C**					
LVIS-0076	NADPH-quinone oxidoreductase	CR	**3.05**	**3.06**	**2.24**
LVIS-0320	NADH dehydrogenase	C	1.457	**2.02**	**2.19**
**COG EF**					
LVIS-0811	ADP-ribose pyrophosphatase	F	1.32	**2.02**	**2.46**
LVIS-0864	Succinyl-diaminopimelate desuccinylase	E	−0.052	1.11	**1.56**
**COG G**					
LVIS-1594	Phosphopentomutase	G	−0.97	1.14	**1.82**
LVIS-1742	L-ribulokinase (putative)	G	**2.13**	0.77	1.00
LVIS-2259	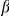 -galactosidase	G	−0.396	1.34	**1.78**
**COG I**					
LVIS-0187	Acetoin reductase	IQ	−0.13	**1.80**	**1.84**
LVIS-0925	Enoyl-(ACP) reductase	I	0.49	**1.91**	**1.82**
LVIS-0926	Acetyl-CoA carboxylase 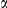 subunit (AccA)	I	0.29	0.54	**1.68**
LVIS-0927	Acetyl-CoA carboxylase 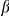 subunit (AccB)	I	1.26	1.33	**1.88**
LVIS-0928	Biotin carboxylase	I	0.77	**1.85**	**2.41**
LVIS-0929	FabZ	I	1.049	**1.79**	**2.23**
LVIS-0930	Biotin carboxyl carrier protein	I	−0.22	1.19	**1.43**
LVIS-0931	3-oxoacyl-(ACP) synthase (FabF)	IQ	0.62	**1.61**	**3.20**
LVIS-0932	3-oxoacyl-(ACP) reductase (FabG)	IQR	0.68	**2.36**	**2.98**
LVIS-0933	(ACP) S-malonyltransferase	I	0.74	**2.15**	**2.76**
LVIS-0934	Acyl carrier protein	IQ	0.36	**2.08**	**2.59**
LVIS-0935	3-oxoacyl-(ACP) synthase III (FabH)	I	**1.55**	**2.23**	**2.62**
LVIS-0937	FabZ	I	1.203	**2.40**	**2.78**
**COG KO**					
LVIS-0112	Molecular chaperone (sHSP)	O	**1.96**	**2.01**	**1.65**
LVIS-0762	ATP-binding subunit of Clp, DnaK	O	**3.51**	**2.78**	**1.69**
LVIS-0809	Peptide methionine sulfoxide reductase	O	1.14	**1.97**	**2.36**
LVIS-0810	Peptide methionine sulfoxide reductase	O	1.01	**1.55**	**2.13**
LVIS-0936	Transcriptional regulator	K	0.91	**2.37**	**2.48**
LVIS-1216	Thiol-disulfide isomerase and thioredoxin	O	1.18	**1.6**	**1.52**
LVIS-1328	DnaJ-like molecular chaperone	O	**1.87**	**2.21**	**1.87**
LVIS-1554	ATP-binding subunit of Clp, DnaK	O	1.50	**1.73**	0.89
LVIS-1700	ATP-binding subunit of Clp, DnaK	O	**1.66**	**1.56**	**1.57**
LVIS-1701	Repressor of class III stress genes	K	**2.01**	**1.63**	**1.56**
LVIS-2091	RNAP sigma subunit, 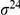 -like	S	0.67	−0.52	**2.25**
**COG PSR**					
LVIS-0116	Cation transport ATPase	P	**1.64**	**1.57**	0.79
LVIS-0471	ABC-type Mn/Zn transporter, ATPase	P	**1.64**	**1.56**	0.69
LVIS-0472	ABC-type Mn 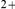 /Zn 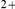 transporter	P	1.44	**1.59**	0.85
LVIS-1844	Aldo/keto reductase	R	**2.30**	**2.85**	**2.24**

COG definitions: C: Energy production and conversion, D-Cell cycle control, cell division, chromosome partitioning, G-Carbohydrate transport and metabolism, I-Lipid transport and metabolism, K-Transcription, O-Posttranslational modification, protein turnover, chaperones P-Inorganic ion transport and metabolism, Q-Secondary metabolites biosynthesis, transport and catabolism, R-General function prediction only, and S-Function Unknown. Each log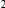 score is bolded if the gene is significantly overexpressed during that time point.

**Table 4:** Expression Levels of Select Significantly Downregulated Genes during 2% n-butanol Stress.

Locus	Gene Description	COG(s)	15 min Log 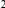	75 min Log 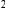	135 min Log 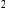
**COG C**					
LVIS-0514	L-lactate dehydrogenase	C	−0.35	−0.24	**−1.49**
LVIS-1558	NAD(FAD)-dependent dehydrogenase	R	**−1.38**	**−1.29**	**−1.52**
LVIS-2211	Na 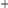 /H 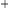 antiporter	C	**−2.07**	**−1.70**	**−1.31**
**COG EJ**					
LVIS-0078	Glutamate 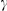 -aminobutyrate antiporter	E	−0.95	**−1.73**	**−1.30**
LVIS-1712	Amino acid transporter	E	0.24	**−1.47**	**−1.18**
LVIS-1781	Amino acid transporter	E	−1.21	**−1.26**	−0.77
LVIS-2023	Carbamate kinase	E	**−2.10**	**−1.93**	**−1.33**
LVIS-2024	Transaminase	E	**−2.52**	**−1.85**	**−1.18**
LVIS-2025	Amino acid transporter	E	**−2.49**	**−1.59**	−0.86
LVIS-2026	Ornithine carbamoyltransferase	E	**−2.66**	**−1.86**	−0.33
LVIS-2027	Arginine deiminase	E	**−3.70**	**−2.04**	−0.89
LVIS-2049	Branched-chain amino acid permease	E	**−1.29**	−1.03	−0.71
LVIS-2213	Glutamate decarboxylase	E	**−2.27**	**−1.78**	**−1.17**
**COG G**					
LVIS-0413	Glycerate kinase	G	−1.07	−1.05	**−1.56**
LVIS-0661	Glyceraldehyde-3-phosphate dehydrogenase	G	**−1.61**	−1.02	**−1.19**
LVIS-0689	Glucosamine-6-phosphate isomerase	G	**−1.71**	**−1.54**	**−1.38**
**COG M**					
LVIS-1047	UDP-N-acetylmuramate-L-alanine ligase	M	−0.82	−1.04	**−1.27**
LVIS-1417	Cell wall-associated hydrolase	M	**−1.78**	−1.11	−0.43
LVIS-1419	Cell wall-associated hydrolase	M	**−1.30**	−0.93	−0.99
LVIS-1496	N-acetylmuramoyl-L-alanine amidase	M	**−1.58**	**−1.27**	**−1.28**
LVIS-1548	Integral membrane protein	S	0.08	−0.96	**−1.18**
LVIS-1575	Glycosyltransferase-like protein	M	**−1.78**	−1.04	−0.84
LVIS-1809	Cell wall-associated hydrolase	M	**−1.48**	−0.33	−0.42
LVIS-2047	Cyclopropane fatty acid synthase-like protein	M	**−1.48**	−1.14	−0.76
**COG S**					
LVIS-1641	Cytochrome bd-type quinol oxidase, subunit 2	C	**−1.30**	−0.89	−0.26

COG definitions: C: Energy production and conversion, D-Cell cycle control, cell division, chromosome partitioning, G-Carbohydrate transport and metabolism, I-Lipid transport and metabolism, K-Transcription, O-Posttranslational modification, protein turnover, chaperones P-Inorganic ion transport and metabolism, Q-Secondary metabolites biosynthesis, transport and catabolism, R-General function prediction only, and S-Function Unknown. Each log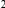 score is bolded if the gene is significantly overexpressed during that time point.

Amino acid, carbohydrate, and ion transport and metabolism are significantly altered during the n-butanol stress response. Several unannotated amino acid transporters are weakly downregulated only in the first initial time point following n-butanol addition (LVIS-0619, LVIS-1789) while no change in the metabolism of amino acids known to function as osmoprotectants [68] is seen. Other metabolic functions involved in amino acid biosynthesis (LVIS-2023-LVIS-2027) are highly repressed as well. The expression of various enzymes responsible for the uptake and metabolism of carbohydrates (particularly pentoses), including phosphopentomutase (LVIS-1594), L-ribulokinase (LVIS-1742), and 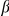-galactosidase (LVIS-2259) continually increases throughout the experiment, perhaps as part of a concerted effort to support the energy demanding stress response. The strong downregulation of a Na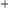/H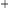 antiporter (LVIS-2211) comports well with the observed effects of n-butanol on H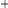 gradients in other organisms [64], [65]. In addition to these metabolic changes, cell wall synthesis is also downregulated immediately following the n-butanol challenge, though this repression lessens over time. While these expression patterns are consistent with a reduced growth rate during an energy-consuming stress response, repression of other proteins possibly involved in competing phenotypes is also seen. The putative homologs of several proteins involved in acid tolerance in *E. coli* including glutamate decarboxylase (LVIS-2213) and glutamate acid resistance system gadC (LVIS-0078) [69], along with cyclopropane fatty acid synthase (LVIS-2047) [70] are downregulated for much of the time series. Other elements of the *Lactobacillus* acid stress response such as the arginine deiminase pathway (LVIS-2023, LVIS-2026, LVIS-2027) [71] are also strongly repressed. These responses are curiously similar to the observed antagonism between ethanol and acid tolerance phenotypes in *E. coli* [72], suggesting that the same effect may exist between the n-butanol and acid tolerance phenotypes.

**Figure 2:**
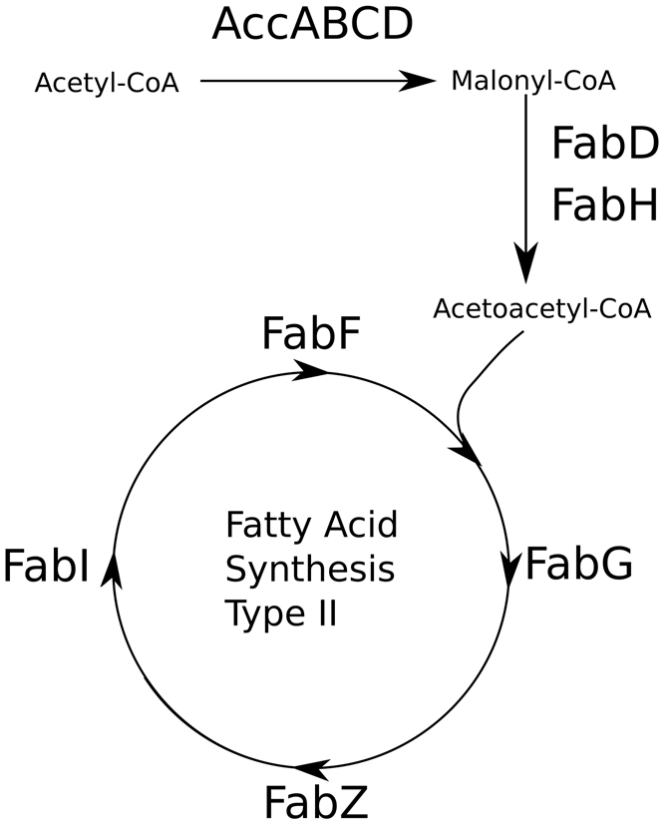
A visualization of the Type II fatty acid synthesis pathway found in most bacteria [**62**]. The AccABCD proteins carboxylate acetyl-CoA to form malonyl-CoA, followed by condensation of malonyl-CoA with acetyl-CoA to form acetoacetyl-CoA by FabH. Other proteins including FabG, FabZ, FabI and FabF elongate the acetoacetyl-CoA by two carbons every pass through the cycle. Transcription of each FAS gene is only slightly upregulated immediately following n-butanol addition but increases significantly after 75 and 135 minutes.

Along with these oxidative and general stress adaptations that are common to most organisms exposed to n-butanol, an upregulation of the entire fatty acid synthesis pathway is clearly evident from the transcriptional data. The FA synthesis pathway itself is summarized in Figure 2. Increased expression of the acetyl-CoA carboxylase subunits (accABCD) shunts additional acetyl-CoA to malonyl-CoA for dedicated use in the FAS pathway [62], [73], [74]. Subsequently, after the initial condensation step by *fabH*, *fabFGZI* homologs identified with BLAST in *L. brevis* (see Table 3) continue the fatty acid elongation process. Following termination, these fatty acids are generally converted into membrane phospholipids by the glycerolphosphate acyltransferase system [75]. The expression of nominal equivalents for the *plsX* (LVIS-0954) and *plsC* (LVIS-1355) do not significantly change over the course of the experiment so it is possible these enzymes are not a rate-limiting step in the membrane lipid synthesis, are regulated post-transcriptionally, or that the fatty acid flux is being diverted elsewhere in the cell. To test whether increasing flux through the FAS pathway affected n-butanol tolerance, the *accABCD*-overexpression strain developed by Davis et al. [74] was subjected to a n-butanol challenge. No significant change of maximum n-butanol tolerance or growth rate was observed with this strain, suggesting the increased production of fatty acids was insufficient or actually unable to confer a protective effect.

n-Butanol and other solvents alter the ratio of saturated and unsaturated membrane lipids in many organisms [44], [45], [76]. Altered gene expression within the fatty acid synthesis regulon may therefore be partially responsible for the n-butanol tolerance *L. brevis*. Direct assessment of the *L. brevis* membrane fatty acid composition following 75 min of 2% (v/v) n-butanol stress revealed only a significant 21.6% decrease in the abundance of 19∶1 cyclopropane fatty acid (19∶1-cfa) compared to unchallenged control cultures (Student's one tailed t-test, 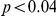). This fatty acid comprises only a small proportion of all fatty acids found in the *L. brevis* membrane during exponential growth, from 5.67% for the unchallenged controls versus 4.46% for the cultures subjected to n-butanol insult. Given the known role of cycloproprane fatty acids in conferring acid tolerance [70], [77], this result supports the inference that n-butanol and acid tolerance are opposing phenotypes, albeit for mechanistic reasons that are unclear. In order to test whether this hypothesis carries over to other organisms, the growth of a *cfa* gene knockout *E. coli* strain in 0–2% n-butanol was compared to the *E. coli* BW25113 parent strain. Unexpectedly, the growth rate and maximum n-butanol tolerance of the strains were very similar; this result could be explained by a lack of cyclopropane fatty acids in the *E. coli* membrane during exponential growth so that knocking out the *cfa* would have little effect on the actual membrane composition. A decrease in cyclopropane fatty acid content is also observed in several *E. coli* strains evolved for isobutanol tolerance [78]. Additional work is needed to unravel the role that cyclopropane fatty acids play in modulating n-butanol resistance and to further investigate the seemingly antagonistic relationship between n-butanol and acid tolerance.

Numerous studies have examined the transcriptional programs of *C. acetobutylicum* and *E. coli* in response to n-butanol challenge to better understand possible tolerance mechanisms. We chose to compare the n-butanol-induced transcriptional responses of *L. brevis* and *E. coli* for this purpose as *E. coli* is a widely used common bacterial host for a variety of applications in the biotechnology industry. The recent study by Rutherford and coworkers (2010) [46] identified several features of the n-butanol stress response in *E. coli*: increased expression of genes involved with oxidative phosphorylation (*nuo*, *cyo*, and *sdh* operons), an oxidative stress response involving *sodA* (superoxide dismutase) and *yqhD* (alcohol dehydrogenase), perturbed amino acid and carbohydrate transport, and an extracytoplasmic stress response as indicated by *cpxP*, *degP*, *spy*, and *rpoE* expression. Though the lack of known functions for many *L. brevis* genes hinders a direct comparison between these organisms, the expression of oxidative stress genes and the disturbances in *L. brevis* metabolism (in terms of transport and energy demands) agree well with those seen in *E. coli*. While *L. brevis* may also be under significant extracytoplasmic stress, the *L. brevis rpoE*-like sigma factor is not significantly upregulated at any time point, though a 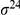-like protein is upregulated during late 2% n-butanol stress. No other regulators are apparent from BLAST comparisons with *E. coli* genes; however, the *dnaJ* and LVIS-0112 chaperones along with several other protein chaperones are upregulated as expected. Strikingly, no statistically significant upregulation of the *E. coli* FAS genes was, in contrast to the constantly increasing expression of FAS genes in *L. brevis* following n-butanol induction.

### Conclusions

This study was aimed at revealing possible components of the *L. brevis* stress response to ferulic acid and n-butanol, two distinct microbial inhibitors of great industrial importance. The results presented here combined with previous studies on the mechanisms of phenolic acid toxicity suggest that alterations in membrane structure and fluidity may play an important role in maintaining cell integrity and physiological electrochemical gradients in *L. brevis* when exposed to ferulic acid. The diverse range of membrane proteins expressed by *L. brevis* are likely involved in ameliorating abnormal ion flux across the membrane while enhancing rigidity, though molecular characterization of the gene products is needed for confirmation. Introduction of efflux pumps to expel ferulic acid and its decarboxylation product along with ion pumps to maintain intracellular K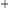, H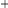, and Na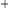 at appropriate concentrations in the presence of ferulic acid may be promising steps towards improving the phenolic acid tolerance of other organisms based upon these results.

Unlike its response to ferulic acid, *L. brevis* responds to n-butanol stress by countering oxidative damage, increasing carbon uptake, altering ion transport, and upregulation of the entire fatty acid synthesis operon. Direct analysis of the *L. brevis* membrane composition also revealed a significant decrease in the abundance of 19∶1 cycloproprane fatty acid. However, the n-butanol tolerance of an *E. coli cfa* knockout was not affected compared to wild-type, indicating additional elements of the stress response are required for tolerance. Both *E. coli* and *C. acetobutylicum* have the same generalized stress response but lack overexpression of the FAS genes, suggesting that increased fatty acid synthesis contributes to n-butanol tolerance in *L. brevis*. The upregulated fatty acid synthesis may act to restore membrane homoeostasis in opposition to n-butanol-induced fluidization. Biochemical characterization of how *L. brevis* regulates this FAS response and the ultimate intracellular source of the fatty acids is crucial to replicating this desirable phenotype in more industrially suitable organisms. The implied competition between acid and alcohol tolerance phenotypes seen in the transcriptional data and fatty acid composition changes suggests that evaluating the n-butanol tolerance of strains with deletions of genes that confer acid resistance may reveal unexpectedly resistant phenotypes.

## Materials and Methods

### Bacterial Strains and Growth Conditions

*L. brevis* ATCC367 (American Type Culture Collection) and *E. coli* BW25113 (CGSC) were used in this study. *L. brevis* cultures were all grown at 30

C and 100 rpm unless otherwise noted. *E. coli* BW25113 *cfa*::*kan* was obtained from the Keio collection [79]. Overnight cultures used to initiate the time series inhibition experiments were grown in baffled 250 ml flasks with 25 ml MRS media (Difco) to 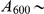
1.5–1.8. Three 500 ml baffled flasks with 125 ml MRS media were then inoculated with 3 ml of overnight each and grown to 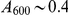 (mid-exponential phase; see Figure S3) for the ferulic acid and n-butanol challenges. Each 125 ml culture of *L. brevis* was challenged with 0.6 g ferulic acid (24 mM) or 1–2% (v/v) n-butanol. Subsequently, culture samples were rapidly harvested by filtration after 15, 75, and 135 minutes to track the immediate stress response and any long-term adaptations to either inhibitor. At each time point, samples were rapidly harvested by filtration using 0.22 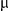
m analytical filters (Nalgene) and placed immediately into 10 ml solution of RNAlater (Ambion) to preserve RNA integrity for later processing. Prior to the addition of ferulic acid, a pooled culture was created, harvested, and stored for use as a reference. Cell samples were stored at −80

C for subsequent analysis. Three biological replicates each were used in this study for the ferulic acid and 1% n-butanol (v/v) stressed cultures, and two biological replicates were used for 2% (v/v) n-butanol stressed cultures.

### Genetic Manipulation

To evaluate the effect of upregulated fatty acid synthesis on *E. coli* n-butanol tolerance, HB101(DE3) [80], kindly provided by Zhilei Chen (Texas A&M University), was transformed with the pMSD8 plasmid containing the AccABCD synthetic operon (generously provided by J. Cronan) [74] via electroporation in a Gene Pulser XL (Bio Rad). The growth rate of the HB101(DE3)/pMSD8 strain was then evaluated in M9 minimal media with 0%–2% n-butanol using an TECAN Infinite M200 plate reader (TECAN) over a period of 24 hours.

### Extraction of Total RNA

Extraction of total RNA was performed with the ZR Fungal/Bacterial RNA MiniPrep kit (Zymo Research Corp) as follows: For each time point, 1.5 ml samples stored in RNALater were pelleted at 16000×g for 20 minutes and the supernatant was removed by aspiration. The bacterial pellet was then processed according to the manufacturer's protocol except that one volume of ice-cold ethanol was used to assist the RNA precipitation. DNAse I treatment was performed in-column as specified by the manufacturer. The resulting RNA was quantified using the Qubit fluorometer (Invitrogen). Gel electrophoresis was also used to confirm RNA quality. If necessary, samples were concentrated using ethanol precipitation and resuspended in 14 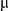
l of molecular biology grade water [81].

### Labeled cDNA Generation and Microarray Hybridization

The SuperScript indirect cDNA labeling system (Invitrogen) was used to generate cDNA incorporating amino-allyl dUTP. Cy3 and Cy5 (GE Healthcare) or Alexa Fluor (Invitrogen) dyes were used to label the cDNA samples. Custom cDNA microarrays (Agilent) containing 15,209 probes (excluding positive and negative controls) were designed using the software package Picky [82] to maximize probe specificity and sensitivity under hybridization conditions with the following parameters. All probes are sixty base pair oligomers with 100% similarity to their corresponding target sequence. The minimum acceptable 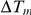 between the probe target and other sequences was set at 15

C, with a GC content range between 30–70%. The salt concentration in the hybridization media was set to 750 mM (personal communication with Agilent). The arrays contain at least three probes per ORF over 100 bp in length (2,157 ORFs total) and 1 probe for every 1050 bp of the *L. brevis* genome [56]. Probes for the 33 protein coding genes located on the *L. brevis* megaplasmids were not included in this study; the majority of these genes are related to plasmid maintenance, mobilization, or partitioning and are therefore not expected to impact the transcriptional data significantly. Labeled cDNA was hybridized onto the cDNA arrays for 18 hours, washed with Agilent Wash Buffers 1 and 2, and then immediately scanned using a GenePix 4200A reader according to the manufacturer's protocol as described for Agilent two-color prokaryotic microarrays.

### Microarray Data Analysis

The local background intensity was subtracted from the recorded signal from each array spot. Arrays were then subjected to LOWESS normalization individually using the MIDAS software package (TM4) [83]. The arithmetic average of the biological and technical replicate sample and reference signals were used for downstream analysis [46]. Differentially expressed (DE) genes were identified for each time point using the rank product method with a critical p-value 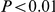 [84]. The currently known functional annotations for *L. brevis* genes were obtained from the US Department of Energy Joint Genome Institute [2], and the number of differentially expressed hypothetical membrane proteins was identified by screening all differentially expressed hypothetical genes with the trans-membrane hidden Markov model TMHMM algorithm [61]. If required, BLAST [56] was utilized to determine *L. brevis*-*E. coli* homologous gene pairs. The statistical significance of the differentially expressed gene functional distributions were assessed using a hypergeometric distribution method [85]. The MeV (TM4) microarray analysis software was used for clustering and other expression profile analysis. The raw microarray data is available from the Gene Expression Omnibus (accession no. GSE24944).

### Fatty Acid Methyl Ester Analysis

*L. brevis* cultures were grown to 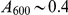 for fatty acid content analysis. Control cultures were pelleted and the supernatant removed immediately, followed by storage at −80

C until processing. Experimental cultures were exposed to 2% n-butanol for 75 min and then stored in an analogous manner. Two biological replicates were used for each condition. Fatty acid analysis was later performed by Microbial ID Inc. to identify statistically significant changes in membrane composition following the n-butanol challenge.

## Supporting Information

Figure S1 **Functional Distribution of Significantly Expressed Genes during Ferulic Acid Stress.** COGs-C: Energy production and conversion, D: Cell cycle control, cell division, chromosome partitioning, E: Amino acid transport and metabolism, F: Nucleotide transport and metabolism, G: Carbohydrate transport and metabolism, H: Coenzyme transport and metabolism, I: Lipid transport and metabolism, J: Translation, ribosomal structure and biogenesis, K: Transcription, L: Replication, recombination and repair, M: Cell wall/membrane/envelope biogenesis, O: Posttranslational modification, protein turnover, chaperones, P: Inorganic ion transport and metabolism, Q: Secondary metabolites biosynthesis, transport and catabolism, R: General function prediction only, S: Function unknown, T: Signal transduction mechanisms, U: Intracellular trafficking, secretion, and vesicular transport, V: Defense mechanisms.
(TIFF)

Figure S2 **Functional Distribution of Significantly Expressed Genes during 2% n-Butanol Stress.** COGs-C: Energy production and conversion, D: Cell cycle control, cell division, chromosome partitioning, E: Amino acid transport and metabolism, F: Nucleotide transport and metabolism, G: Carbohydrate transport and metabolism, H: Coenzyme transport and metabolism, I: Lipid transport and metabolism, J: Translation, ribosomal structure and biogenesis, K: Transcription, L: Replication, recombination and repair, M: Cell wall/membrane/envelope biogenesis, O: Posttranslational modification, protein turnover, chaperones, P: Inorganic ion transport and metabolism, Q: Secondary metabolites biosynthesis, transport and catabolism, R: General function prediction only, S: Function unknown, T: Signal transduction mechanisms, U: Intracellular trafficking, secretion, and vesicular transport, V: Defense mechanisms.
(TIFF)

Figure S3
**Growth kinetics of** ***L. brevis*** **in the absence of inhibitors.** Ferulic acid and n-butanol were added to the cultures at OD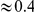 (mid-exponential phase).(TIFF)

Table S1
**Statistically significant log** 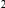 **gene expression values for** ***L. brevis*** **cultures responding to ferulic acid stress.**
(XLSX)

Table S2
**Statistically significant log** 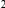 **gene expression values for** ***L. brevis*** **cultures responding to 2% n-butanol stress.**
(XLSX)

Table S3
**Statistically significant log** 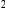 **gene expression values for** ***L. brevis*** **cultures responding to 1% n-butanol stress.**
(XLSX)
