# Supplementary figures and images for: Transcriptional Analysis of Lactobacillus brevis to N-Butanol and Ferulic Acid Stress Responses
Source: PLoS One. 2011 Aug 2;6(8):e21438. doi: 10.1371/journal.pone.0021438 (PMC3149049; doi:10.1371/journal.pone.0021438)

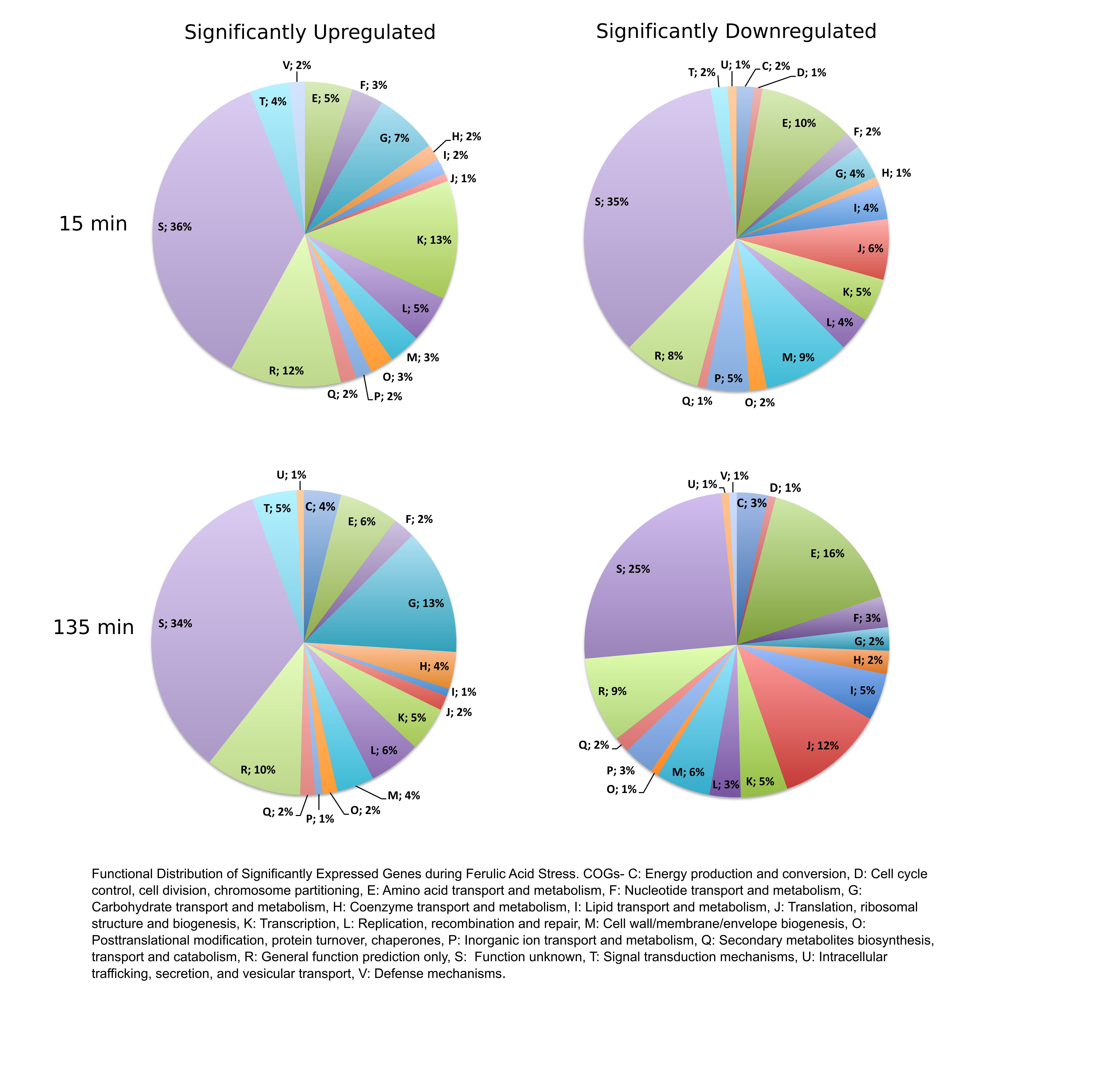

Supplement: Figure S1 — Functional Distribution of Significantly Expressed Genes during Ferulic Acid Stress. COGs-C: Energy production and conversion, D: Cell cycle control, cell division, chromosome partitioning, E: Amino acid transport and metabolism, F: Nucleotide transport and metabolism, G: Carbohydrate transport and metabolism, H: Coenzyme transport and metabolism, I: Lipid transport and metabolism, J: Translation, ribosomal structure and biogenesis, K: Transcription, L: Replication, recombination and repair, M: Cell wall/membrane/envelope biogenesis, O: Posttranslational modification, protein turnover, chaperones, P: Inorganic ion transport and metabolism, Q: Secondary metabolites biosynthesis, transport and catabolism, R: General function prediction only, S: Function unknown, T: Signal transduction mechanisms, U: Intracellular trafficking, secretion, and vesicular transport, V: Defense mechanisms. (TIFF) [file pone.0021438.s001.tiff]

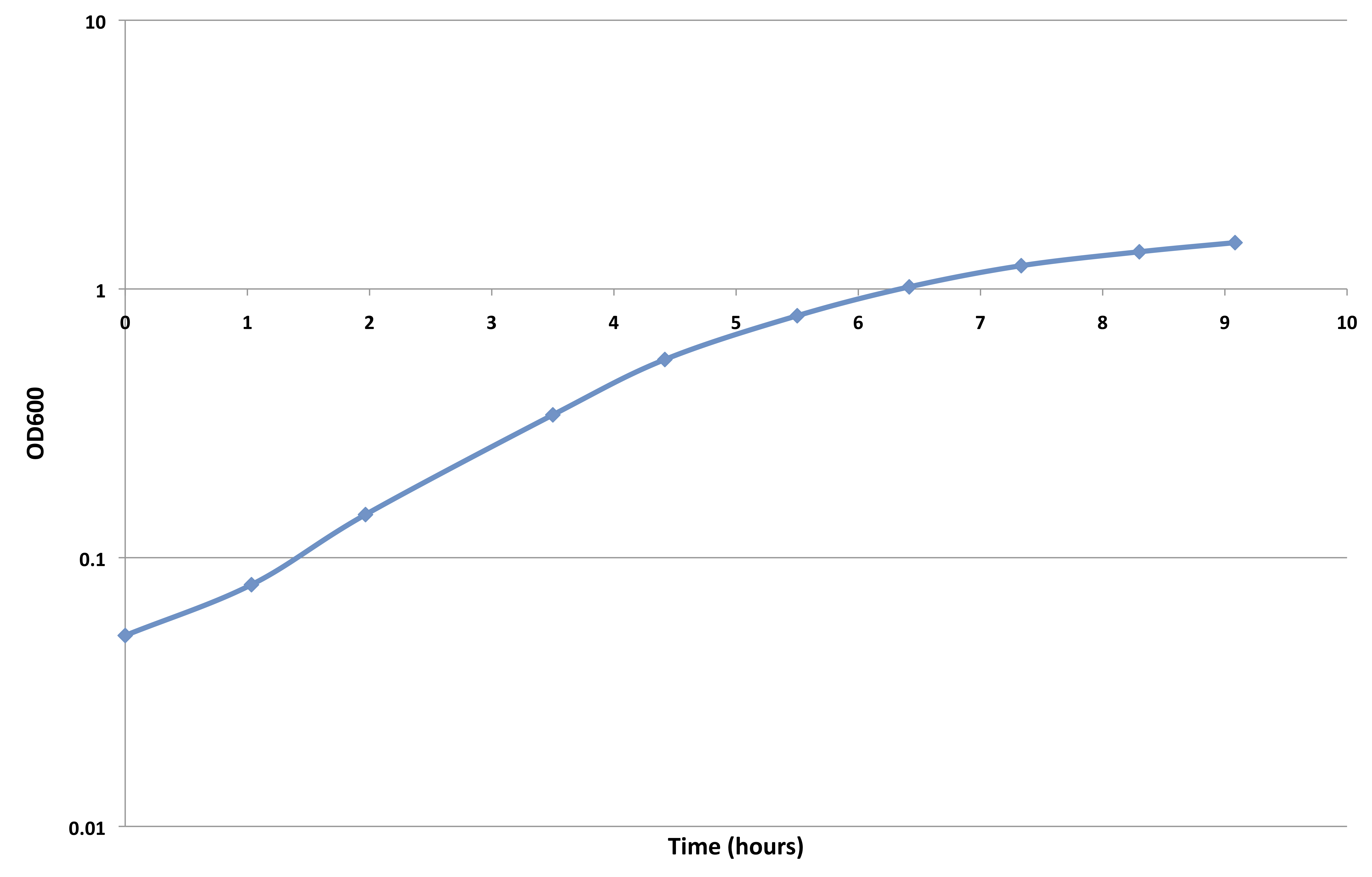

Supplement: Figure S3 — Growth kinetics of L. brevis in the absence of inhibitors. Ferulic acid and n-butanol were added to the cultures at OD (mid-exponential phase). (TIFF) [file pone.0021438.s003.tiff]
